# Mesenchymal Stromal Cell Immunology for Efficient and Safe Treatment of Osteoarthritis

**DOI:** 10.3389/fcell.2020.567813

**Published:** 2020-09-22

**Authors:** Mehdi Najar, Johanne Martel-Pelletier, Jean-Pierre Pelletier, Hassan Fahmi

**Affiliations:** Osteoarthritis Research Unit, University of Montreal Hospital Research Center, Department of Medicine, University of Montreal, Montreal, QC, Canada

**Keywords:** mesenchymal stromal cells, therapeutic effects, immunity, tissue repair, safety, efficiency

## Abstract

Mesenchymal stem cell (MSC) therapy represents a promising approach for the treatment of osteoarthritis (OA). MSCs can be readily isolated from multiple sources and expanded *ex vivo* for possible clinical application. They possess a unique immunological profile and regulatory machinery that underline their therapeutic effects. They also have the capacity to sense the changes within the tissue environment to display the adequate response. Indeed, there is a close interaction between MSCs and the host cells. Accordingly, MSCs demonstrate encouraging results for a variety of diseases including OA. However, their effectiveness needs to be improved. In this review, we selected to discuss the importance of the immunological features of MSCs, including the type of transplantation and the immune and blood compatibility. It is important to consider MSC immune evasive rather than immune privileged. We also highlighted some of the actions/mechanisms that are displayed during tissue healing including the response of MSCs to injury signals, their interaction with the immune system, and the impact of their lifespan. Finally, we briefly summarized the results of clinical studies reporting on the application of MSCs for the treatment of OA. The research field of MSCs is inspiring and innovative but requires more knowledge about the immunobiological properties of these cells. A better understanding of these features will be key for developing a safe and efficient medicinal product for clinical use in OA.

## Introduction

Recent advances in stem cell research have highlighted the role played by these cells and their environment in tissue homeostasis. Several resources of cells can be used to restore the damaged tissue, such as resident stem cells, multipotent adult progenitor cells or embryonic stem cells (Sánchez et al., 2012). As a cell-based therapy product, stem cells have created great hope among the medical field due to their therapeutic potential. However, there are ethical and safety concerns regarding the clinical use of human embryonic stem cells (hESC) and induced pluripotent stem cells (iPSC) (Volarevic et al., 2018). As relatively free from ethical concerns and safer, mesenchymal stem cell (MSCs) are a valuable alternative for cell-based therapy. Their ease of isolation and high *ex vivo* expansion potential have allowed a broad use of MSCs (Bianco et al., 2008).

Mesenchymal stem cells display a specific immunological profile and functions allowing them to efficiently down-regulate immunoinflammatory events and to promote tissue regeneration. In case of tissue injury, local tissue precursor cells with immunomodulatory capacities were described to be recruited and activated (Hoogduijn, 2015).

Initially, the therapeutic effects of MSCs were thought to be mediated based on their multilineage differentiation ability that enabled them to replace damaged cells in injured tissue. Nonetheless, the capacity of MSCs to transdifferentiate into *tissue*-specific cell types *in vivo* has not been fully confirmed. This is because it is hard to track MSCs after transplantation due to the lack of reliable MSC-specific markers *in vivo*. Subsequent findings indicate that MSCs promote tissue repair through the production of a myriad of trophic factors, including growth factors, chemokines, cytokines and anti-oxidants, rather than direct differentiation and cell replacement (Becerra et al., 2011; Damia et al., 2018; Harrell et al., 2019b; Jimenez-Puerta et al., 2020).

This has prompted the development of numerous preclinical studies as well as clinical trials that demonstrated promising therapeutic results (Noronha et al., 2019). In some cases, the benefits of MSCs are not satisfactory and need to be improved (Andrzejewska et al., 2019; Rendra et al., 2020).

In this review, we mainly focused on the biological effects of MSCs upon their transplantation. We described the characteristics of the transplantation, the immune and blood compatibility, which are relevant for the therapy outcome. Following transplantation, MSCs modulate the local tissue homeostasis and immune responses (Nolta et al., 2020). It should be noted that MSCs do not need to migrate to injured tissue in order to exert their regenerative and immunomodulatory effects. For instance, intraperitoneal injection of allogenic MSCs reduced the severity of cartilage and bone damage in collagen-induced arthritis independently of MSC migration to the joints (Augello et al., 2007). Similarly, Swart et al. (2015) showed in a mouse model of proteoglycan-induced arthritis that intraarticular injection of MSCs ameliorates systemic responses independently of their capacity to migrate from the site of injection. These data suggest that MSCs may exert their beneficial effects on distant tissues, likely via extracellular vesicles.

A clear identification of the crosstalk between MSCs and the immune cells present within the tissue environment as well as their role during tissue repair is required. We therefore reported on the latest advances regarding the main functions and mechanisms of action of MSCs, considering the influence of the tissue microenvironment. In particular, we focused on the cellular and molecular changes that may affect MSCs (i.e., cell death) and contribute to these therapeutic effects. Improving our understanding of the immunological profile and therapeutic effects of MSCs will help to develop a safe, feasible, and efficient cell-therapy strategy.

## The Characteristics of the Transplantation

The transplantation of therapeutic cells depends on both donor and recipient specificities to guide the selection of a suitable graft. Several parameters including the type of transplantation (i.e., autologous, allogeneic, and xenogeneic), the route of cell delivery as well as the blood and immune compatibility of the cellular product should be examined before performing the therapy (Patrikoski et al., 2019).

### Surface Phenotype of MSCs

The International Society for Cell Therapy (ISCT) has defined MSCs with a minimal set of three standard criteria: (a) adherence to plastic under standard culture conditions, (b) expression of CD105, CD73, and CD90, and lack expression of CD45, CD34, CD14 or CD11b, CD79α, or CD19, and HLA-DR surface molecules, and (c) differentiation into osteoblasts, adipocytes, and chondrocytes *in vitro* (Dominici et al., 2006).

However, these markers are not specific to undifferentiated MSCs and are also detected in other cell types such as fibroblasts and smooth muscle cells (Samsonraj et al., 2017). In addition, MSCs are a heterogeneous population of cells with varying degrees of self-renewal capacity and differentiation potential. Therefore, other surface antigens including CD10, CD13, CD29, CD44, CD49, CD54, and CD166 (Samsonraj et al., 2017) are also used considered as MSC markers (Table 1). Recently, the ISCT recommended that the acronym MSCs should be accompanied by tissue-source origin which would feature tissue-specific properties (Viswanathan et al., 2019).

**TABLE 1:** Cell surface positive and negative markers of human MSCs as derived from Samsonraj et al. (2017).

**Positive markers**	**Negative markers**
CD105	CD45
CD73	CD34
CD90	CD14
HLA-ABC	CD11
CD10	CD79
CD13	CD19
CD29	HLA-DR
CD44	CD40
CD49	CD80L
CD54	CD80
CD166	CD86

### The Immunological Profile of MSCs

The immunologic profile of MSCs has revealed that they express low levels of major histocompatibility complex (MHC) class I molecules. They do not express MHC class II molecules and co-stimulatory molecules CD40, CD80 and CD86, which participate in T cell activation.

This particular immunophenotypic profile allows MSCs to escape immune surveillance and promotes their hypoimmunogenic or immune privileged status. MSCs do not elicit a proliferative response when cocultured with allogeneic T cells *in vitro*. However, some studies reported that MSCs may express these molecules and lose their hypoimmunogenic/immune privileged state. For example, treatment with interferon gamma (IFNγ), which represents an inflammatory environment, induces the expression of MHC class II molecules and increases the expression of MHC class I molecules (Romieu-Mourez et al., 2007; Van Megen et al., 2019).

As mentioned previously, MSCs typically do not express HLA-DR, an MHC class II molecule which plays important roles in allograft rejection. However, MScs may express these molecules during cell expansion (Grau-Vorster et al., 2019) and thus fail to meet all the ISCT’s requirements for MSCs. MSCs were also reported to express HLA-DR after differentiation. For instance, Ryan et al. (2014) demonstrated that chondrogenically differentiated MSCs express HLA-DR. Chondrogenic MSCs induce the proliferation of both CD4 and CD8 cells and increase susceptibility to cytotoxic lysis by allospecific T cells. Moreover, they lose their immunosuppressive properties as evidenced by their inability to prevent T cell proliferation. Subcutaneous implantation of chondrogenically differentiated MSCs increased the infiltration of mononuclear phagocytes and the generation of anti-donor IgG2 antibodies (Ryan et al., 2014). This raises the concern that after differentiation or transplantation, MSCs trigger immune responses, which may hamper their therapeutic efficacy. Further studies are clearly needed to determine the impact of HLA-DR expression in chondrogenically differentiated MSCs on their therapeutic efficacy in OA.

### Blood Compatibility of MSCs

The ABO blood group is one of the major immunogenic barriers hampering tissue transplantation into immunocompetent hosts. Indeed, incompatible blood group antigens are highly immunogenic and can cause graft rejections. Such issues may be instrumental in better defining their therapeutic potential in clinical trials. MSCs do not present carbohydrate- and protein-based membrane structures that are defined as blood group antigens. Moreover, MSCs do not upregulate ABO blood group antigens after inflammatory challenge or *in vitro* differentiation confirming that their therapeutic efficacy is not altered by immunogenic blood group antigens (Schäfer et al., 2011).

### Selection of Autologous or Allogeneic Transplantation

For cell therapy purposes, the use of *in vitro*-expanded autologous or allogeneic cell populations is possible. In autologous transplantation, the cells are collected from the patient’s own tissues, which does not require human leucocyte antigen (HLA) matching therefore avoiding immunological complications (Champlin, 2003). However, allogeneic transplants between two genetically different individuals may be associated with some difficulties (Liras, 2010). Indeed, the graft type of MSCs is a key determinant for the success of the therapy because it is closely linked to the immune response that may be elicited by the recipient. While most of these clinical and preclinical trials utilized autologous MSCs, a significant number of studies examined the feasibility of allogeneic or even xenogeneic MSC transplantation (Lin et al., 2012).

The use of autologous MSCs is time-consuming and costly with additional drawbacks such as donor site morbidity and quality issues in patients with comorbidities or advanced age whose MSCs may have reduced therapeutic efficacy. In contrast, allogeneic MSCs appear to be one of the most promising candidates for therapeutic applications because it provides “off-the-shelf” cellular therapy (Wang et al., 2019). Consequently, understanding their interactions with the recipient’s immune system is crucial for their successful clinical application (Kot et al., 2019). Importantly, evidence from currently completed and ongoing clinical trials demonstrate that allogeneic MSC transplantation is safe and seems to cause no major side effects to the patient (Kot et al., 2019). For instance, the POSEIDON clinical trial provided evidence that allogenic MSCs display superior efficacy to autologous MSCs in the treatment of non-ischemic dilated cardiomyopathy (Hare et al., 2017). Interestingly, allogenic MSCs were shown to promote cartilage regeneration and improve the symptoms of OA in two recent clinical trials (Vega et al., 2015; de Windt et al., 2017).

Transplantation of xenogenic MSCs is often unsuccessful due to irreconcilable interspecies differences. There is phylogenetic distinction based on differences in the key mediators of the immunosuppressive effects of MSCs (Su et al., 2014). Moreover, differences in cytokine signaling might lead to failure of MSC activation and therefore to therapeutic misinterpretation or lack of *in vivo* efficient effect. Thus, interspecies incompatibilities from preclinical data should be taken into consideration before translation to clinical trial (Lohan et al., 2018). Overall, the characterization of a functional population of MSCs with a specific profile and function may ultimately influence the choice between autologous or allogeneic transplantation.

### The Delivery Route of MSCs

Depending on the clinical purposes, MSCs are administered differently, either systemically infused, locally injected, or locally applied in a cell-carrier glue (de Windt et al., 2017). The optimal cell delivery technique should provide the most regenerative benefit with the lowest side effects. The most common routes of MSC transplantation outside tissue engineering-based methods are by intravenous or intra-arterial infusion, or by direct intra-tissue injection (Kurtz, 2008). Local transplantation deposits MSC in spatial proximity to the lesion, i.e., intraarticularly in the case of OA. Systemic administration routes are favored but require the targeted extravasation of the circulating MSCs at the site of injury. Transplanted MSCs can indeed leave the blood flow and transmigrate through the endothelial barrier, and reach the lesion site (Nitzsche et al., 2017).

## The Therapeutic Effects of MSCs

The beneficial effects of MSCs rely mostly on their capacity to sense tissue injury, and consequently to display several coordinated therapeutic actions. Through their regulatory and trophic factors, MSCs attenuate detrimental immune response, remove pathogens, and promote the functions of local cells (Harrell et al., 2019a). We highlighted hereafter some relevant elements that contribute to the therapeutic process of MSCs.

### The Process of Tissue Injury and Healing

The physiological response to tissue damage involves three consecutive and coordinated phases: inflammatory, reparative, and remodeling. During this process, the inflammatory/immunological status (defined as the nature of immune cells as well as the types and concentrations of present cytokines) varies considerably (Wang et al., 2014). During the first phase of healing, there is a predominance of proinflammatory signals which decrease in the reparative and remodeling phases (wound healing period). The prevalence of proinflammatory mediators induce the recruitment of inflammatory cells (such as neutrophils, monocyte, and platelets). Monocytes/macrophages play the leading role in innate immunity and tissue homeostasis. These cells accumulate in site of injury and are actively involved in tissue repair (Wallace et al., 2012). Then, the infiltrated neutrophils begin to undergo apoptosis, which causes macrophages to shift toward an anti-inflammatory phenotype (wound-healing subset).

### MSCs Are Environmentally Responsive

The dynamic flux in the immune microenvironment is essential to facilitate the migration and proliferation of therapeutic cells to repair and regenerate tissue (Toh et al., 2018). Depending on the signals sensed by MSCs, they can migrate and home within a specific tissue. Indeed, MSCs are sensitive to shifts in the local milieu as they harbor a panel of receptors activating various signaling pathways (Paladino et al., 2019). We have previously shown that MSCs express several relevant receptors, such as the receptor for advanced glycation end-products (RAGE), C-type lectin receptors (CLRs, including DECTIN-1, DECTIN-2 and MINCLE), leukotriene B4 (LTB4) receptors (BLT1 and BLT2) and cysteinyl leukotriene (CysLTs) receptors (CYSLTR1 and CYSLTR2) (Najar et al., 2018). These receptors, known for their role in the regulation of inflammatory and immunological responses, were significantly modulated following MSC exposure to inflammatory signals. It is now recognized that the functions of MSCs are not constitutive but induced during their presence in the injured site (Kaundal et al., 2018). This plasticity in their properties allows MSCs to acquire specific phenotypes and functions.

### Mobilization and Homing of MSCs

Mesenchymal stem cells reside in their tissue in normal physiological conditions but seem to have the capacity to be mobilized in response to signals produced by injured tissues. These signals may have a role in determining the function of MSCs, e.g., in the promotion of pathogen clearance or the modulation of the inflammation. In response to local environmental cues, MSCs start circulating, proliferate, and migrate from their niche to the injury site. The homing of MSCs is based on a multistep model involving (1) initial tethering by selectins, (2) activation by cytokines, (3) arrest by integrins, (4) diapedesis or transmigration using matrix remodelers, and (5) extravascular migration toward chemokine gradients (Ullah et al., 2019). MSC migration *in vitro* can be induced by different growth factors and chemokines and is enhanced by the pro-inflammatory cytokine tumor necrosis factor alpha TNF-α (Ponte et al., 2007), suggesting that the mobilization of MSCs and their subsequent homing to injured tissues may depend on the systemic and local inflammatory state. Moreover, under injury conditions, endothelial cells are activated and express docking molecules such as CD106 and CD62E (E-selectin). Their ligands, CD49d/CD29 (integrin α4/β1) and CD44, respectively, are expressed by MSCs and are important for their homing and docking (Rüster et al., 2006). In line with this, we have shown that the expression of adhesion molecules by MSCs are tightly regulated and differentially modulated depending on the cell environment. Specifically, we found that an inflammatory or infectious environment, as well as an activated immune response lead to a significant increase of CD54 (intercellular adhesion molecule 1) and CD58 (lymphocyte function-associated antigen 3) expression (Najar et al., 2010). These data were further corroborated by the evidence that damage/inflammatory mediators initiate a cascade of endothelial and leukocyte/MSC adhesion and motility responses relevant to the repair process (Nitzsche et al., 2017). These findings indicate that the homing and adhesion of MSCs are substantially sensitive to the local environment with the injured tissue and are therefore decisive for their therapeutic functions.

### MSC-Mediated Cell Empowerment

Initially, the popular appeal as cell-based therapy was based on the *in vitro* multilineage potential of MSCs. Indeed, the tissue repair capability of MSCs was thought to be consecutive to their local differentiation into functional cells to replace the damaged cells. However, there is no *in vivo* evidence that these cells exert their regenerative effects through engraftment and differentiation into target cells (Ayala-Cuellar et al., 2019). In addition, the lack of standardized methods for their isolation, expansion, and identification does not allow to define terminally differentiated and functionally mature populations (Nombela-Arrieta et al., 2011). It has been demonstrated that the multipotency of MSCs is not a pivotal aspect of cell therapy, and thus primarily referred to their paracrine function as a major activity in tissue repair (Drela et al., 2019). In fact, the tissue−specific resident cells of the patient are actively involved in tissue regeneration and repair. These processes are stimulated by the bioactive factors secreted by the exogenously supplied MSCs, rather than by direct differentiation of MSCs (Prockop, 2007). Consequently, upon arrival at damaged tissue, MSCs are believed to exert their regenerative and repair effects by cell “empowerment” rather than by cell replacement. It is likely that MSCs regulate the local environment during tissue repair and provide a good “soil” for tissue regeneration.

It is increasingly recognized that the local environment with it stromal and immunological components (both cellular and molecular) are significantly important for the success of the therapy (Li H. et al., 2019). Indeed, the therapeutic effect of MSCs is mainly a combination of immunomodulation and local cell “empowerment” (Wang et al., 2014). The inhibition of local inflammation and immune responses (immunomodulation) by MSCs establishes a favorable environment to initiate tissue regeneration through empowering the activities of local tissue stem/progenitor cells. A concerted action of secreted factors by MSCs will induce tissue repair through promoting angiogenesis, remodeling of the extracellular matrix, stimulating the proliferation and differentiation of progenitor/resident cells, and the recruitment of endogenous stem cells to the site of engraftment (Qi et al., 2018). Moreover, several studies underline bioactive exchanges, including ions, nucleic acids, proteins, and organelles transferred from MSCs to stressed cells, thereby improving cell survival and/or renewal in damaged or diseased tissues (Naji et al., 2019).

### The Antimicrobial Activity of MSCs

Tissue injury may be also accompanied by infection due to pathogen invasion which may delay the healing process. In this context, MSCs were shown to have strong antimicrobial activities exerted through indirect and direct mechanisms. Most of the data on the antimicrobial properties of MSCs have been obtained from *in vitro* studies with bacteria, although little data exist about the effect of MSCs on viruses, fungi, and parasites. For instance, MSC administration to dogs with spontaneous chronic multi-resistant wound infections led to bacterial clearance and wound healing (Johnson et al., 2017). These effects are partially mediated by the secretion of antimicrobial peptides and proteins (AMPs) (Alcayaga-Miranda et al., 2017). Depending on the tissue origin of MSCs, several AMPs such as cathelicidins (e.g., LL-37), β-defensins (hBD-1, hBD-2, and hBD-3), hepcidin, or lipocalin families (e.g., Lcn2) have been described. These AMPs represent the major arm of the innate immunity and play important roles in initiating inflammation and further immune responses. Moreover, they participate in wound repair by stimulating the expression of cytokines and chemokines involved in the recruitment of immune cells and tissue progenitors (Chow et al., 2020).

### Therapeutic Effects of MSCs in OA

**FIGURE 1:**
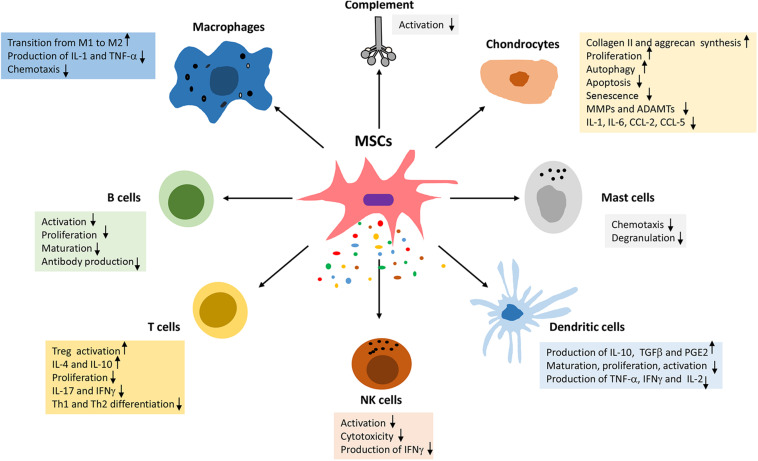
Immunomodulatory effects of MSCs in osteoarthritis.

Mesenchymal stem cells have been used for the treatment of OA based on their chondrogenic potential or their ability to promote cartilage repair through stimulation of endogenous cells and immunomodulation. In addition MSCs have significant paracrine activity, whereby they secrete a wide array of growth factors, cytokines, and chemokines that mediate various effects on chondrocytes including stimulation of proliferation, autophagy, and ECM synthesis (anabolic activity), as well as the inhibition of apoptosis, senescence, and the production of pro-inflammatory and catabolic factors (Figure 1) (for reviews, see references Damia et al., 2018; Harrell et al., 2019b).

In recent years, an increasing number of studies have suggested that the beneficial effects of MSCs are primarily mediated by extracellular vesicles (EVs), particularly exosomes. Cosenza et al. (2017) showed that the treatment of murine OA-like chondrocytes with BM-MSC-derived exosomes promoted anabolic activities (type II collagen and aggrecan), inhibited catabolic (MMP-13 and ADAMT-5) and inflammatory (iNOS) responses, and protected from apoptosis. Using a collagenase-induced OA mouse model, they reported that intraarticular injection of BM-MSC-derived exosomes prevented both cartilage and bone damage. Qi et al. (2019) demonstrated that treatment of rabbit chondrocytes with BM-MSC-derived exosomes prevented IL-1-induced apoptosis, likely via inhibition of the p38 and ERK MAPKs and activation of the Akt pathway. Inhibition of apoptosis by MS-MSC-derived exosomes was also reported in rat chondrocytes (Zhu et al., 2018). More recently, He et al. (2020) evaluated the effect of BM-MSC-derived exosomes on inflammatory and catabolic responses *in vitro* and on the progression of OA in a rat model of the disease. They found that treatment with exosomes diminished the inhibitory effect of IL-1 on the proliferation, migration, and anabolic activity of chondrocytes. Accordingly, *in vivo* studies revealed that administration of exosomes was protective *in vivo*, likely via increased anabolic responses and reduced catabolic responses in the joints (He et al., 2020).

AT-MSC-derived exosomes were also reported to have chondroprotective properties. Treatment of human OA chondrocytes with AT-MSC-derived exosomes decreased IL-1-induced production of numerous inflammatory and catabolic mediators including TNF-α, IL-6, PGE2, NO and MMP13, whereas the production of the anti-inflammatory cytokine IL-10 and type II collagen were enhanced (Tofiño-Vian et al., 2018). The expression of COX-2 and mPGES1 were also down-regulated. These changes were likely due to reduced activity of NF-kB and AP-1. Similarly, Zhao et al. (2020) reported that AT-MSC-derived exosomes displayed chondroprotective and anti-inflammatory properties. They found that co-culture of AT-MSC-derived exosomes with activated synovial fibroblasts reduced the expression of IL-6 and TNF-α, whereas the expression of IL-10 was enhanced. Co-culture with chondrocytes protected from H_2_O_2_-induced apoptosis. Interestingly, treatment with exosomes stimulated chondrogenesis and increased the expression of chondrogenic markers, such as collagen type II and β-catenin (Zhao et al., 2020). More recently, it was evidenced that human AT-MSC-derived EVs increased human OA chondrocyte proliferation and migration, enhanced type II collagen synthesis, and reduced IL-1-mediated expression of key catabolic enzymes, MMP-1, MMP-3, MMP-13, and ADAMTS-5 (Woo et al., 2020). Further *in vivo* studies indicated intraarticular injection of AT-MSC-derived EVs attenuated cartilage degradation and synovial inflammation in both monosodium iodoacetate-induced OA in rats and destabilization of the medial meniscus (DMM)-induced OA in mice (Woo et al., 2020).

In addition to bone marrow and adipose tissue, exosomes isolated from other sources such as embryonic stem cells have also shown beneficial effects in cartilage repair and OA. For instance, Zhang et al. (2016) demonstrate that human embryonic MSC-derived exosomes promote cartilage repair and regeneration in a rat model of osteochondral defects. After 12 weeks, exosome-treated defects displayed complete recovery of hyaline cartilage characterized by regular biosynthesis and deposition of type II collagen and glycosaminoglycan (GAG). Using a mouse model of instability-induced OA, Wang and colleagues showed that human embryonic MSC prevented cartilage erosion and the expression of ADAMTS-5, a key enzyme in cartilage degradation. Moreover, *in vitro* experiments revealed that treatment with MSC-derived exosomes preserved chondrocyte phenotype upon treatment with IL-1β (Wang et al., 2017).

## Immunoregulation as a Key Mechanism in Tissue Repair

As previously evoked, MSCs promote tissue repair and regeneration through cell-empowerment and favoring an immune tolerogenic environment. Indeed, MSCs are not immune cells but regulatory progenitors with strong immunomodulatory properties. They can interact with different types of immune cells, leading to reciprocal interplay and modulation (Nemeth, 2014). MSC-mediated immunomodulation operates through a synergy of cell contact-dependent mechanisms and release of soluble factors (Li Y. et al., 2019). These pathways, as it will be highlighted below, cooperate to create a tolerogenic environment suitable for tissue regeneration.

### Recruitment of Regulatory Immune Cells

Mesenchymal stem cells can interact with various types of immune cells, including T cells, B cells, natural killer (NK) cells, macrophages, dendritic cells, neutrophils, and monocytes (Leyendecker et al., 2018a). After these interactions, several features linked to immune response such as activation, proliferation and functions of immune cells are modulated by MSCs. We and others have reported that several regulatory immune cells such as Treg, Breg, NKreg, M1/M2, and DCreg are generated from both the innate and adaptive responses following contact with MSCs (Najar et al., 2016). These regulatory cells accumulate within the tissue of interest and regulate the local immune environment to facilitate the tissue repair.

### Production of Immunoregulatory Mediators

Both direct cell–cell contact (membrane bounded proteins and receptors) and secretion of regulatory mediators can underline the immunomodulatory effects of MSCs. The secretome of MSCs is composed of cytokines, chemokines, and trophic factors that can be released in the extracellular milieu or within EVs (Zhou et al., 2019). Many mediators were shown to contribute to the therapeutic effects of MSCs including transforming growth factor (TGF)-β1, hepatocyte growth factor (HGF), prostaglandin E2 (PGE_2_), indoleamine 2,3-dioxygenase (IDO), nitric oxide (NO), leukemia inhibitory factor (LIF), HLA-G, heme oxygenase-1 (HO-1), insulin growth factor (IGF)/IGF- binding protein (BP) system, TNF-a-stimulated gene 6 (TSG-6), metalloproteinases (MMP-2; MMP-9), TIMP-2 tissue inhibitor of metalloproteinases (TIMP-2; TIMP-3) and chemokine (C-C motif) ligand 2 and 5 (CCL2; CCL5), interleukin (IL)-10, IL-6, semaphorins, galectins, CD200/CD200R, erythropoietin-producing hepatocellular (Eph) receptor tyrosine kinase-B/Eph family receptor interacting proteins (ephrin)-B, glycoprotein A repetitions predominant (GARP), and purinergic signaling (Damia et al., 2018; Harrell et al., 2019b).

IL-10, a pleiotropic immunomodulatory cytokine, modulates both the innate and adaptive immune systems. Interestingly, several regulatory factors produced by MSCs, such as HGF, TSG-6, PGE2, IDO, HLA-G, and LIF, closely interact with IL-10 to establish a tolerogenic milieu suitable for T-cell inhibition. In addition, there are several interplays between IL-10 and these factors including reciprocal positive feedback loops. IL-10 seems to be primarily derived from immune cells, in particular T cells, and demonstrates an increased level during interactions with MSCs. In this context, we demonstrated that the IL-10/CD210 axis is critical during immunomodulation by inducing proliferative and molecular changes within the immune cells (Najar et al., 2015). Recently, a dose-dependent transfer of mitochondria (MitoT) by MSCs was suggested to promote Treg differentiation, which may rescue target organs from tissue damage and inflammatory response (Court et al., 2020). Additional mediators including lipids, messenger RNAs (mRNAs) and microRNAs (miRNAs) can also contribute to the therapeutic effects of MSCs through their pro-angiogenic, antifibrotic, antiapoptotic or anti-inflammatory properties (Pers et al., 2018a).

### Regulation of Metabolic Pathways

There is a close link between the metabolism of immune cells and their biological features. Several metabolic pathways are considered as important actors for regulating immune responses. Indeed, immune cell activation, differentiation, and function require specific energetic and biosynthetic demands (Patel et al., 2019). Metabolic fitness has been shown to be crucial for supporting the major shift from quiescent to active immune cells and for tuning the immune response. Recent studies have shed new light on the role of the end products of metabolism such as lactate, acetate, and adenosine triphosphate (ATP) (Degauque et al., 2018). Such products are likely to participate to tissue and immune homeostasis and are therefore important during transplantation. Intracellular ATP is well-known as the energy source driving cell survival, proliferation, and metabolic function (Pearce and Pearce, 2013). However, under tissue stress, ATP can be released from cells into the extracellular environment. In that sense, MSCs were shown to modulate the immune response by a dynamic ATP hydrolysis (Burr and Parekkadan, 2019). ATP was shown to promote the immunosuppressive properties of MSCs via upregulation of IDO expression (Lotfi et al., 2018). It is noteworthy that, the level of ATP should be well controlled since uncontrolled levels can affect several cellular features and functions. In the case of human endometrium MSCs, ATP was shown to induce cell cycle arrest, alter the proliferative and migration capacity and therefore could affect their regenerative potential (Semenova et al., 2020).

Adenosine (ADO) is a nucleoside with pleiotropic functions, which acts as an intracellular and extracellular mediator of multiple biological processes, including immune responses. It is considered as a common path for MSCs and Treg-mediated immunosuppression (De Oliveira Bravo et al., 2016). In fact, the production of adenosine constitutes a mechanism used by both cell types to control the immune response particularly in the inflammatory environment. To produce ADO, ATP is hydrolyzed to 5′-AMP and ADP by the ectonucleotidase CD39. ADP is further hydrolyzed to ADO by the second ectonucleotidase CD73. Although CD73 is one of the main and highly expressed markers within MSCs, the expression and modulation of CD39 by MSCs has also been confirmed (Kerkelä, 2017). Whereas MSCs from different tissues exhibit many common characteristics, their biological activity and some markers are different and depend on their tissue of origin. Changes in the expression profile of certain markers is also dependent on the environment surrounding MSCs (Kozlowska et al., 2019). Current data indicate that MSCs exhibit different sensitivity to purinergic ligands as well as a distinct activity and expression profiles of ectonucleotidases than mature cells. MSCs may abundantly produce ADO in contrast to other progenitor cells (Jeske et al., 2020). The adenosinergic pathway emerges as a key mechanism by which MSCs exert hemostatic and immunomodulatory functions. Depending on the CD73/adenosine pathway, MSCs inhibited platelet activation and aggregation (Netsch et al., 2018), altered T-cell activation (Chen et al., 2016), and reduced NK cell activity (Yan et al., 2019). Of note, NK cells interacting with MSCs may acquire the expression of external nucleotide CD73. These new CD73−positive NK cells can regulate the function of resting NK cells in either an autocrine or paracrine manner. Intriguingly, the inhibition of CD39 and CD73 ectonucleotidases enhanced the mobilization of MSCs by decreasing the extracellular level of adenosine, which may influence the therapeutic outcomes (Adamiak et al., 2019). The heterogeneity in CD73 expression and its catalytic products may have distinct modulatory effects on the local immune response. This statement may explain the differences observed during tissue regenerative cell-based therapy (Tan et al., 2019).

Overall, it is important to consider and revise the influence of immunometabolism on the therapeutic process of MSCs. This will improve our understanding of the immunobiology of MSCs as well as their therapeutic efficacy.

### Immunomodulatory Properties/Effects of MSCs in OA

Osteoarthritis has long been considered a “wear and tear” disease culminating in cartilage loss, but it is now widely accepted that inflammation plays a key role in its pathogenesis. The inflammatory cycle of OA is thought to result from interactions between the immune system and local tissue degradation products. Accumulating clinical evidence recognizes synovial inflammation (synovitis) as a characteristic of OA (Robinson et al., 2016). It is present in about half of the patients with OA and has been shown to correlate with the severity of knee OA symptoms, particularly pain (Hill et al., 2007) and with cartilage damage severity (Ayral et al., 2005).

In OA, synovial membranes are infiltrated with various immune cells predominantly monocytes/macrophages followed by T cells. Mast cells, NK cells, dendritic cells, B cells and granulocytes have also been identified in OA synovium. This topic has been more comprehensively reviewed elsewhere (van den Bosch et al., 2020).

MSCs have significant immunomodulatory capacity and can suppress all immune cells involved in the development and progression of OA (Figure 1). MSCs can promote macrophage transition from the IL-1 and TNF-α producing pro-inflammatory M1 phenotype to the IL-10, IL-RA, and TGF-β producing anti-inflammatory and pro-chondrogenic phenotype (Fernandes et al., 2020). The effect of MSCs on macrophage polarization are mediated via TNFα-stimulated gene/protein 6 (TSG-6), prostaglandin E2 (PGE2) and indoleamine 2,3-dioxygenase (IDO) (Fernandes et al., 2020).

Mesenchymal stem cells, can suppress the proliferation and function of CD4+ and CD8+ T cells and promote the proliferation of immunosuppressive T regulatory cells (Luque-Campos et al., 2019). Moreover, MSCs prevent most functions of NK cells including cytotoxicity, cytokine and granzyme B secretion (Luque-Campos et al., 2019). MSCs were also shown to inhibit the proliferation of autoreactive B cells (de Castro et al., 2019). The proliferation, maturation, and antigen-presenting function of dendritic cells (DC) are also suppressed by MSCs (Spaggiari et al., 2009). Moreover, MSCs suppress numerous functions of mast cells including degranulation, cytokine production and chemotaxis (Brown et al., 2011). Further studies showed that MSC inhibit activation of the complement system (Tu et al., 2010), which plays a central role in the pathophysiology of OA.

Last but not least, MSCs or exosomes may induce their protective effects in OA by modulating chondrocyte functions. Specifically, MSCs were reported to prevent several inflammatory and catabolic events in chondrocytes and cartilage explants (Harrell et al., 2019b). Reports also showed that MSCs enhance chondrocyte proliferation, autophagy and the synthesis of cartilage extracellular matrix (Harrell et al., 2019b).

## Cell Death as a Component of MSC Immunomodulatory Properties

Once transplanted, MSCs may face a harsh microenvironment such as hypoxia, oxidative stress, damage signals, inflammatory, and immunological reactions. Such environments may blunt their engraftment, viability, and functionality indicating that there are further mechanisms by which MSCs repair tissue. It appears that the secretome is only one part of MSC effects, as the viability of MSCs does not appear to be a prerequisite for some of their therapeutic effects (Naji et al., 2019). Different cellular and molecular alterations underlining distinct cell death modes are observed during tissue regeneration (Liu et al., 2018).

### Apoptosis

Apoptotic MSCs have been shown to participate in the tissue repair process and immunomodulation (Weiss and Dahlke, 2019). These findings are in keeping with the “dying stem cell hypothesis” stating that the apoptosis of MSCs causes a modulation of the local immune response with a down-regulation of the innate and adaptive immunity (Thum et al., 2005). Usually, apoptosis is an immunologically quiescent process dependent on normal numbers of apoptotic cells (ACs) and rapid clearance by professional and non-professional phagocytes within the injured tissue. MSCs were reported to directly phagocyte ACs, therefore increasing their PGE_2_ production, which contributes to MSC-based immunotherapeutic effects (Zhang et al., 2019). In turn, under certain conditions, living MSCs may be subject to perforin-induced apoptosis through recipient cytotoxic cells (CTL or NK cells). Apoptotic MSCs could then face phagocytosis by host-innate immune cells (monocytes/macrophages). Thus, the roles of both “being eaten” and “eating others” appear to be implicated in the immunomodulation mechanisms of MSCs (Zhang et al., 2019). Indeed, apoptotic, metabolically inactivated, or even fragmented MSCs have been shown to possess an immunomodulatory potential as well (Weiss and Dahlke, 2019). After phagocytosis of MSCs, monocytes are polarized toward an immunoregulatory M2 phenotype and redistributed systemically. This mechanism may explain how MSCs with reduced life induce long lasting immunomodulatory effects (Weiss et al., 2019).

Mesenchymal stem cell efferocytosis (phagocytic clearance of apoptotic cells) has also been reported to contribute to their immunomodulatory effects (Piraghaj et al., 2018). Apoptotic MSCs release “find-me” signals that recruit macrophages which recognize “eat-me” signals such as phosphatidylserine (PtS). This recognition triggers an actin-mediated cytoskeletal rearrangement that enables engulfment of the apoptotic MSCs by macrophages. Efferocytosis culminates by the clearance of the dying/dead cells and their toxic components as well as the expression of immune tolerance factors (Galipeau and Sensébé, 2018). Recently, MSCs have been demonstrated to harness macrophage derived amphiregulin (AREG) to maintain tissue homeostasis after injury. By increasing the secretion of AREG in a phagocytosis-dependent manner, MSC-primed macrophages allowed immunosuppression through the promotion of regulatory T (Treg) (Ko et al., 2020).

### Complement Mediated Cell Death

In addition to its role in the innate immune system, the complement pathway can contribute to tissue repair at different levels (Schraufstatter et al., 2015). The complement cascade may stimulate the phagocytosis of pathogens and damaged cells but also the recruitment of stem and progenitor cells to the site of injury. In parallel, it promotes inflammation and adaptive immune response as well as activation of cell death pathway. There is an interplay between the complement-mediated cell lysis and distinct cell death pathways (Fishelson et al., 2001). Activation of the terminal pathway of the complement system leads to insertion of terminal complement complexes (C5b-9) into the cell membrane, which may induce apoptosis via a caspase-dependent pathway. Apoptosis as a consequence of complement-mediated cell damage may provide an explanation for the presence of apoptosis in inflammatory processes, for instance in hyperacute xenograft rejection (Nauta et al., 2002). The complement system has been shown to interact with MSCs and to differentially influence some of their biological features (Schraufstatter et al., 2009). Accumulating evidence suggests that molecules of the innate immune system, including complement components and pentraxins, have a role in the recognition and clearance of apoptotic cells (Nauta et al., 2003). In a complement-activated environment, MSCs are injured following formation of membrane attack complexes (MACs), which may be linked to the rapid clearance of systemically circulating MSCs after infusion (Li and Lin, 2012). Moreover, complement-mediated opsonization has a pivotal role in immune tolerance by recognition and uptake of apoptotic cells and modulation of cytokine release (Jin and He, 2017). It was reported that complement activity, by binding to MSCs, promotes their phagocytosis by monocytes, which may shift into M2-healing subsets thus contributing to establishing a tolerogenic environment (Gavin et al., 2019). Intriguingly, MSCs have been shown to express complement inhibitor proteins CD46, CD55, CD59, and Factor H suggesting that they are partially protected from the lytic activity of complement (Tu et al., 2010). Indeed, BM-MSCs have been reported to express the receptors for anaphylatoxins C3a and C5a which are highly present within inflamed and injured tissues. Such expression was linked to homing of MSCs to site of injury, resistance to oxidative stress and apoptosis as well as inhibition of immune response (Le Blanc and Davies, 2015). Despite the rapid clearance of MSCs after systemic infusion, a favorable therapeutic effect is still observed. It is possible that complement activation by promoting monocyte phagocytosis of MSCs, participates in tissue repair.

### Autophagy

The therapeutic potential of MSCs may also be linked to autophagy. Autophagy is a highly conserved cellular process that degrades modified, surplus, or harmful cytoplasmic components by sequestering them in autophagosomes, which then fuses with the lysosome for degradation. As a major intracellular degradation and recycling pathway, autophagy is crucial for maintaining cellular homeostasis, as well as for remodeling during normal development (Chen et al., 2018). MSCs may modulate autophagy of tissue-resident and recruited cells (target cells) involved in disease pathogenesis. MSCs can affect autophagy of immune cells involved in injury by reducing their survival, proliferation, and function and favoring the resolution of inflammation. In addition, MSCs can affect autophagy in endogenous adult or progenitor cells, promoting their survival, proliferation and differentiation and thus supporting the restoration of functional tissue (Ceccariglia et al., 2020). Stress signals or pharmacological agents can also modulate autophagy in MSCs. All these types of autophagy may affect MSC functions and have an impact on the therapeutic potential (either directly or indirectly) by influencing survival, vascularization, immunomodulation, and cell differentiation (Jakovljevic et al., 2018).

### Senescence

Successful MSC therapy needs a prolonged and large-scale cell culture which may lead to cell senescence. Administration of senescent MSCs may result in an inefficient therapeutic issue (Li et al., 2017). Therefore, it is of utmost importance to enhance our knowledge of the aging process and methods to detect cell senescence in order to overcome this challenge. Senescence is a cellular response to stress limiting proliferation of damaged and aged cells. It is involved not only in pathological processes but also in physiological mechanisms like aging, tissue repair, and homeostasis (Neri and Borzì, 2020). Several factors such as DNA damage, telomere shortening, oncogenic insults, metabolic stress, epigenetic changes, and mitochondrial dysfunction might induce senescence (Neri and Borzì, 2020). Aging of MSCs (both *in vivo* and *in vitro*) can affect distinct properties of MSCs such as self-renewal, proliferation, differentiation, and immunomodulation thus possibly compromising their therapeutic effect (Chen et al., 2018). A recent study indicated that aging significantly altered distinct biological characteristics of MSCs, with old MSCs displaying reduced proliferation, differentiation potential, immunoregulatory, and secretory ability (Yang et al., 2020).

## The Therapeutic Effect of MSCs Depends on Their Origin

Since their first isolation BM, other alternative tissue sources of MSCs were identified. Because of this diversity, it is important to define MSCs and recognize the inherit differences between these sources (Le Blanc and Davies, 2018). The accessibility, frequency, and properties of MSCs may thus differ, requiring more attention in the choice of the source of MSCs (Busser et al., 2015). Moreover, it is essential to find non-invasive cell sources to avoid donor site morbidity (Pinheiro et al., 2019). In addition to BM, MSCs have also been isolated from adipose tissue, synovial membrane, fetal tissues, and dental pulp (Leyendecker et al., 2018b). MSCs from different tissue sources may share similar phenotypes and proliferation properties, but show distinct transcriptome and cytokine profiles (Meng et al., 2019). Indeed, these MSCs may present unique gene expression pattern that reflects an advantage in terms of biological activities (Alhattab et al., 2019). Several differentially expressed genes were identified among these types of MSCs playing roles in immunomodulation, angiogenesis, wound healing, apoptosis, and chemotaxis (Barrett et al., 2019). These specific signaling pathways suggest that MSCs preserve different functional potentials according to their origin (Najar et al., 2019). For example, synovial and infrapatellar fat pad-derived stem cells present improved proliferative and survival potential in comparison to BM (Fernandes et al., 2018). Wharton’s jelly of the human umbilical cord (WJ-MSCs) were shown to display distinct immunomodulatory and pro-regenerative transcriptional signature compared to BM-MSCs (Donders et al., 2018). WJ-MSCs may thus be considered as potent tolerogenic tools to modulate local immune response in support type regenerative medicine approaches (Corsello et al., 2019). Of note, MSCs are a composite of cell progenitors at different states of lineage commitment and cellular aging (O’Connor, 2019). Recently, several types of oral MSCs have been described as immunomodulatory masters because of their ability to interact with an inflammatory microenvironment and to exert a multitude of immunological actions (Zhou et al., 2020). Moreover, several distinct subpopulations of MSCs with differentially expressed genes related to proliferation, development, and inflammation response were observed in WJ-MSCs (Sun et al., 2020). These subpopulations of MSCs may display distinct tissue repair effects, and therefore represent relevant sources for specific therapeutic applications.

## Clinical Trials Using MSCs for the Treatment of Knee OA

The use of MSCs in the treatment of OA is an expanding and growing area of research, and several studies have reported on the clinical efficacy of MSCs in OA. As stated above, MSCs can be isolated from many different tissues; however, BM- and *adipose tissue-derived MSCs* are the two most commonly used types of MSCs in OA therapy.

Orozco et al. (2013) studied 12 patients with knee OA who received an intraarticular injection of autologous expanded BM-derived MSCs (40 × 10^6^ cells). These patients had Kellgren-Lawrence grade II-IV. They reported that patients had significant improvements in patient reported outcome measures, including visual analog scale (VAS), and the Western Ontario and McMaster Universities Arthritis Index (WOMAC) pain scores at 12 months. Patients also had improved quality of life as assessed by the 36-item Short Form *Health Survey* (*SF*-*36*). Magnetic resonance imaging (MRI) quantitative T2 mapping revealed an improvement of cartilage quality and a decrease of poor cartilage areas (Orozco et al., 2013). These improvements were maintained at 2 years (Orozco et al., 2014).

In a 5-year follow-up study, Davatchi and colleagues investigated the effects of transplanting autologous BM-MSCs in four patients with moderate to severe knee OA. At 6 months post-injection, three patients had improved functions as assessed by reduced walking distance to onset of pain. The number of stairs they could climb and the pain on the VAS were improved for all four patients. Then, they observed a progressive gradual deterioration, but at 5 years the outcomes were still better than at baseline, suggesting a protective role of BM-MSCs compared to untreated controls (Davatchi et al., 2011, 2016). It is noteworthy that this study only included four patients making it difficult to draw firm conclusions.

Lamo-Espinosa and colleagues tested the efficacy of two doses (10 or 100 × 10^6^ cells) of autologous BM-derived MSC in combination with hyaluronic acid (HA) in a randomized controlled clinical trial. Thirty patients with OA (Kellgren–Lawrence grades II–IV) were enrolled with a follow-up period of 12 months. Patients who received BM-MSC showed improvement in WOMAC and VAS pain scores. Accordingly, the range of motion was also improved. Interestingly, radiological and MRI analyses revealed that only high dose treated-patients had significant improvement in cartilage thickness (Lamo-Espinosa et al., 2016). The observed clinical and functional improvement of knee OA was sustained after a follow up of 4 years.

In a similar study Soler et al. (2016), evaluated the effect of autologous BM-MSCs (40.9 × 10^6^ cells) in 15 OA patients (Kellgren–Lawrence grades II–III). Outcomes assessed included VAS for pain, algofunctional Health Assessment Questionnaire, Quality of Life (QoL) SF-36 questionnaire, Lequesne functional index, WOMAC score, and cartilage structure. The authors reported improvements in pain and function, and noted signs of cartilage regeneration at 12 months, which were maintained for 4 years (Soler et al., 2016).

Administration of allogenic MSCs also led to significant improvements in knee OA. In a randomized controlled trial, Vega and colleagues compared the efficacy of allogenic BM-MSCs (40 × 10^6^ cells) to HA in 30 patients. Outcomes analyzed included pain, disability, quality of life and cartilage quality. Compared to HA-treated patients, allogeneic-BM-MSC-treated patients showed improvement in pain and function. Additionally, there was a significant decrease in poor cartilage areas in MSC-treated patients (Vega et al., 2015). The therapeutic effect of observed in this trial was smaller than those reported for autologous MSCs. Further studies comparing the efficacy of autologous with allogenic BM-MSC in the same clinical trial are needed to confirm these findings.

In a randomized, double-blind, controlled trial, Gupta et al. (2016) evaluated the efficacy of different doses of allogenic BM-MSCs (25, 50, 75, or 150 × 10^6^ cells) in 60 OA patients. Outcomes including VAS, WOMAC, intermittent and constant OA pain (ICOAP), and cartilage structure were evaluated at regular intervals for 12 months. All subjective outcomes tended to improve in participants who received MSCs, with the 25 × 10^6^ dose being the most effective. However, MRI evaluation revealed no perceptible change in cartilage structure and integrity (Gupta et al., 2016).

More recently, Chahal et al. (2019), treated 12 patients with escalating doses of autologous BM-MSCs (1, 10, or 50 × 10^6^ cells). There was an overall improvement in pain, symptom, quality of life, and stiffness scores. Best clinical and radiological responses were obtained in patients who received the high dose MSC. Interestingly, the synovial levels of monocytes/macrophages and IL-12 were decreased after MSCs administration. In addition, MSC-treated patients displayed lower cartilage catabolic biomarkers, suggesting a chondroprotective effect of MSCs (Chahal et al., 2019).

Adipose tissue-derived MSCs (AT-MSCs) have also been shown to have beneficial effects in the treatment of OA (Jo et al., 2014; Pers et al., 2016, 2018b). Pers and colleagues evaluated the impact of three doses of AT-MSCs (2, 10, or 50 × 10^6^ cells) in 18 OA patients. The parameters assessed were pain and function. They reported that participants who received low dose of MSCs had the best response in terms of pain and function (Pers et al., 2016). A later study by the same group found that injection of AT-MSCs in the knee triggers a systemic long-lasting immune modulation involving an increase in the percentage of CD4^+^CD25^high^CD127^low^FOXP3^+^ regulatory T cells and CD24^high^CD38^high^ transitional B cells (Pers et al., 2018b).

In a distinct study using similar number of patients, Jo et al. (2014) tested the efficacy of increasing doses of AT-MSCs (10, 50, or 100 × 10^6^ cells). Outcomes included pain, function and cartilage structure. Treatment with either dose improved all algofunctional indices and structural outcomes but statistical significance was reached only with the high dose of MSCs (Jo et al., 2014). It should be noted that the clinical improvement does not last longer and started to decline within 2 years following treatment.

## Conclusion and Perspectives

The utilization of MSCs in the treatment of OA is a promising avenue. There are clearly several cellular regulatory pathways involved in the therapeutic effect of MSCs. These pathways cooperate to promote cartilage regeneration and an anti-inflammatory environment. Moreover, the broad cellular and molecular changes that accompany MSC apoptosis, autophagy, and senescence may be essential for their therapeutic effects. Identifying the function and mode of action of these different cell death pathways will help in improving the efficacy of MSCs in the treatment of OA. From our point of view, two important steps need to be developed to guarantee a successful anti-OA therapeutic strategy based on MSCs:

*The first step* is the understanding of the immunological profile and functions of MSCs as a graft. This would allow to match the adequate needs with the right response. Accordingly, we must find specific immunological signatures that identify these specific therapeutic progenitors.

*The second step* is the understanding of the mechanisms involved in the effects of MSCs for better therapeutic targeting. We should well-understand the tissue injury environment and mechanisms of the recipient that may critically influence the beneficial effects of MSCs.

Collectively, all these features are relevant for developing MSCs as a therapeutic option for OA with high quality, safety and efficiency standards.

## Author Contributions

All authors listed have made a substantial, direct and intellectual contribution to the work, and approved it for publication.

## Conflict of Interest

The authors declare that the research was conducted in the absence of any commercial or financial relationships that could be construed as a potential conflict of interest.
